# Antibody responses to *Bordetella pertussis* and other childhood vaccines in infants born to mothers who received pertussis vaccine in pregnancy – a prospective, observational cohort study from the United Kingdom

**DOI:** 10.1111/cei.13275

**Published:** 2019-03-13

**Authors:** T. F. Rice, D. A. Diavatopoulos, G. P. Smits, P. G. M. van Gageldonk, G. A. M. Berbers, F. R. van der Klis, G. Vamvakas, B. Donaldson, M. Bouqueau, B. Holder, B. Kampmann

**Affiliations:** ^1^ Section of Paediatrics, Department of Medicine Imperial College London UK; ^2^ Section for Pediatric Infectious Diseases, Laboratory of Medical Immunology, Radboud Institute for Molecular Life Sciences Radboud University Medical Center Nijmegen the Netherlands; ^3^ Radboud Center for Infectious Diseases Radboud University Medical Center Nijmegen the Netherlands; ^4^ Centre for Infectious Disease Control National Institute for Public Health and the Environment (RIVM) Bilthoven the Netherlands; ^5^ Department of Biostatistics, Institute of Psychology, Psychiatry and Neuroscience King’s College London UK; ^6^ The Vaccine Centre London School of Hygiene and Tropical Medicine London UK; ^7^ Vaccines and Immunity Theme MRC Unit The Gambia at LSHTM Fajara The Gambia

**Keywords:** antibodies, human, reproductive immunology, vaccination

## Abstract

The maternal Tdap (tetanus, diphtheria and acellular pertussis) vaccination programme in the United Kingdom has successfully reduced cases of pertussis in young infants. In addition to prevention of pertussis cases, it is also important to investigate the persistence of maternal antibodies during infancy and the possible interference of maternal antibodies with infant responses to vaccines. We recruited mother–infant pairs from vaccinated and unvaccinated pregnancies and measured concentrations of immunoglobulin (Ig)G against pertussis toxin (PTx), filamentous haemagglutinin (FHA), pertactin (Prn), diphtheria toxin (DTx), tetanus toxoid (TTx) *Haemophilus influenzae* type b (Hib) and *Streptococcus pneumoniae* in mothers and infants at birth, and in infants at 7 weeks and at 5 months. Thirty‐one mother–infant pairs were tested. Tdap‐vaccinated women had significantly higher antibody against Tdap antigens, compared to unvaccinated women (DTx, *P* = 0·01; PTx, FHA, Prn and TTx, *P* < 0·001). All antibodies were actively transferred to the infants (transfer ratio  > 1) with higher transfer of DTx (*P* = 0·04) and TTx (*P* = 0·02) antibody in Tdap‐vaccinated pregnancies compared to unvaccinated pregnancies. Infants from Tdap‐vaccinated pregnancies had significantly elevated antibodies to all antigens at birth (*P* < 0.001) and at 7 weeks (FHA, Prn, TTx, *P* < 0·001; DTx, *P* = 0.01; PTx, *P* = 0·004) compared to infants from unvaccinated pregnancies. Infants from Tdap‐vaccinated and ‐unvaccinated pregnancies had comparable antibody concentrations following primary pertussis immunization (PTx, *P* = 0·77; FHA, *P* = 0·58; Prn, *P* = 0·60; DTx, *P* = 0·09; TTx, *P* = 0·88). These results support maternal immunization as a method of protecting vulnerable infants during their first weeks of life.

## Introduction

Pertussis is a highly contagious infection of the upper respiratory tract caused by the bacterium *Bordetella pertussis* 1. Although pertussis affects all age groups, complications and mortality from infection are highest in infants too young to be fully immunized. The resurgence of pertussis in vaccinated populations has caused many infant deaths, resulting in a major worldwide public health concern 2. Following 14 infant deaths in the United Kingdom in 2012, a nationwide pertussis vaccination programme for pregnant women was introduced 3. The rationale of maternal vaccination is to boost the observed low pertussis antibody levels in the pregnant population 4, thereby increasing levels of antibodies transferred to the fetus *in utero*. The programme in the United Kingdom is safe 5 and highly effective 6, with the highest proportional reduction in cases and hospital admissions in infants less than 3 months of age 7. Maternal pertussis vaccination has been introduced by the United States, Australia, South American and other European countries 8, 9, 10.

Following acellular pertussis vaccination during pregnancy, antibody concentrations in cord blood to vaccine antigens, including pertussis toxin (PTx), filamentous haemagglutinin (FHA) and pertactin (Prn), are increased in the infant, and to concentrations greater than or equal to those in the mother 11, due presumably to active transport of antibodies across the placenta 12. Associations between high maternal antibody levels in the infant and subsequent impaired vaccine responses have been observed for influenza and measles 13, 14, and recent studies have suggested that maternal pertussis vaccination may be associated with blunted infant responses to primary immunization 15, 16, 17, 18. Since the introduction of the programme, no UK study has reported vaccine responses in mother–infant pairs from vaccinated pregnancies, compared to unvaccinated controls collected during the same time‐period. Our study thus aimed to determine the impact of maternal pertussis vaccination on infant antibody responses to primary immunization with acellular pertussis, *Haemophilus pneumonia* type b (Hib) and *Streptococcus pneumonia *conjugate polysaccharide vaccines.

## Materials and methods

### Study subjects

Women with singleton, uncomplicated term pregnancies booked for maternity care at Imperial College Healthcare NHS Trust were recruited antenatally. Exclusion criteria included maternal autoimmune disease, hypertension, diabetes and pregnancy pathologies. Randomization into vaccinated and unvaccinated groups was not possible for ethical reasons, as the maternal pertussis vaccination programme was in place at the start of the study. The recruits gave birth between May 2014 and September 2016 inclusive. The study was approved by Research Ethics Committee (13/LO/1712) and written informed consent was obtained.

### Serum collection

Maternal serum was routinely collected at time of booking for antenatal care, from the cord immediately at birth and from women postnatally within 72 h of delivery. Serum was collected from infants at 7 weeks (1 week prior to commencing primary immunizations) and 5 months of age (1 month after completion of primary immunizations). Maternal blood collected at the time of booking for antenatal care was taken into Vacutainer^®^ plastic SSTTM II Advance tubes (Becton Dickinson, Wokingham, UK) and stored samples were obtained following patient consent. All other samples were taken into Z Serum Sep Clot Activator tubes (Greiner Bio‐One, Stonehouse, UK) and processed by the study team. Samples were left for a minimum of 30 min prior to centrifugation at 1900 ***g*** for 10 min. Maternal and cord blood were processed within 48 h of collection and infant blood within 1 h of collection. All serum aliquots were stored at –80°C prior to further analysis.

### Vaccines

In line with UK vaccine policy, vaccinated women received tetanus, diphtheria and pertussis‐containing vaccines (Tdap); Repevax^®^ (Sanofi Pasteur, Lyon, France; prior to July 2014) or Boostrix‐IPV^®^ (GlaxoSmithKline, Wavre, Belgium; after July 2014). As per routine vaccination schedules in the United Kingdom, infants received three doses of tetanus, diphtheria and pertussis‐containing vaccine at 8, 12 and 16 weeks; DtaP5‐IPV‐Hib (Pediacel^®^; Sanofi Pasteur) or DtaP3‐IPV‐Hib (Infanrix‐IPV‐Hib^®^; GlaxoSmithKline). All infants received two doses of 13‐valent conjugate pneumococcal polysaccharide vaccine, Prevenar13^® ^(Pfizer, Puurs, Belgium) at 8 and 16 weeks.

### Antibody measurement by multiplex immunoassay

A multiplex immunoassay (MIA) was employed to measure antibody concentrations against pertussis, diphtheria, tetanus, pneumococcal and Hib vaccine antigens at the Centre for Infectious Disease Control, National Institute of Public Health and the Environment (RIVM), the Netherlands. This assay utilizes antigen‐conjugated microspheres to quantify immunoglobulin (Ig)G antibodies using Luminex xMAP technology. Three separate assays were performed as previously described to measure antibodies against: (1) protein antigens PT, FHA, Prn, DT and TT 19, (2) pneumococcal polysaccharide antigens 1, 4, 5, 6B, 7F, 9V, 14, 18C, 19F and 23F 20 and (3) the Hib polysaccharide antigen 21. In brief, standard, control and serum samples were mixed with microspheres conjugated to vaccine antigen proteins, and incubated for 30–45 min. R‐Phycoerythrin‐conjugated goat anti‐human IgG (Jackson ImmunoResearch Laboratories Inc., Westgrove, PA, USA) was added to detect bound antibodies. Samples were processed using a Bio‐Plex 200, and results analysed with Bio‐Plex Manager software version 6.1 (Bio‐Rad Laboratories, Hercules, CA, USA).

### Statistical analysis

The primary end‐points of the study were the determination of antibody levels to pertussis and pneumococcal vaccine antigens at the five study time‐points, stratified by immunization status in pregnancy. Based on previous antibody studies in pregnant women at a single time‐point and assuming 95% protection in vaccinated women and normal distribution of concentrations between the two groups of women, a sample size of 23 per group would theoretically be sufficient to show a significant difference between vaccinated and unvaccinated women with a power of 90%, using a two‐sided test with a significance level of < 0·05.

Results below the limit of detection were assigned the lower limit of quantification: 1 IU/ml for PT, FHA and Prn; 0·001 IU/ml for DT and TT; and 0·01 µg/ml for Hib and all pneumococcal polysaccharide antigens. Appropriate parametric/non‐parametric tests were used following testing for Gaussian distribution using the D’Agostino–Pearson omnibus normality test. Distribution of measures and effects of potential outlying values were examined 22. Outcomes symmetrized by log‐transformation were analysed using mixed‐effects linear regression with a random intercept at the participant level (mixed command in Stata); χ^2^ or *t*‐tests identified baseline characteristics for which the treatment group was not balanced. We estimated the effect of vaccination on antibody concentrations via interactions between the treatment group and time, adjusting variables for groups which were not balanced at baseline or follow‐up. Marginalization was used to present group differences of each time‐point (by use of the contrast and margins post‐estimation commands in stata). Results were adjusted for gestation at delivery. Comparisons of longitudinal antibody concentrations and the effect of gestation at time of maternal vaccination were performed using stata version 15.

The proportion of infant samples with DTx and TTx antibodies ≥0·1 IU/ml 23, and with PTx, FHA and Prn antibodies ≥20 IU/ml 24 were calculated. Comparisons of transfer ratios and antibody half‐life between vaccinated and unvaccinated groups were made using the Mann–Whitney *U*‐test in GraphPad Prism version 7. *P*‐values less than 0·05 were considered significant.

## Results

### Study population demographics

We included a total of 150 serum samples, collected from 31 mother–infant pairs, with 16 obtained from Tdap‐vaccinated pregnancies and 15 from unvaccinated pregnancies. At the 5‐month time‐point, five samples could not be obtained in the unvaccinated group, as mothers withdrew consent for further sampling. Detailed clinical data and a study flow diagram are shown in Supporting information, Table S1 and Fig. S1, respectively. There were no significant demographic nor clinical differences between vaccinated and unvaccinated mother–infant pairs apart from higher parity in unvaccinated mothers, which was corrected for in the analyses.

### Robust maternal antibody responses to Tdap booster vaccination in pregnancy

**Figure 1:**
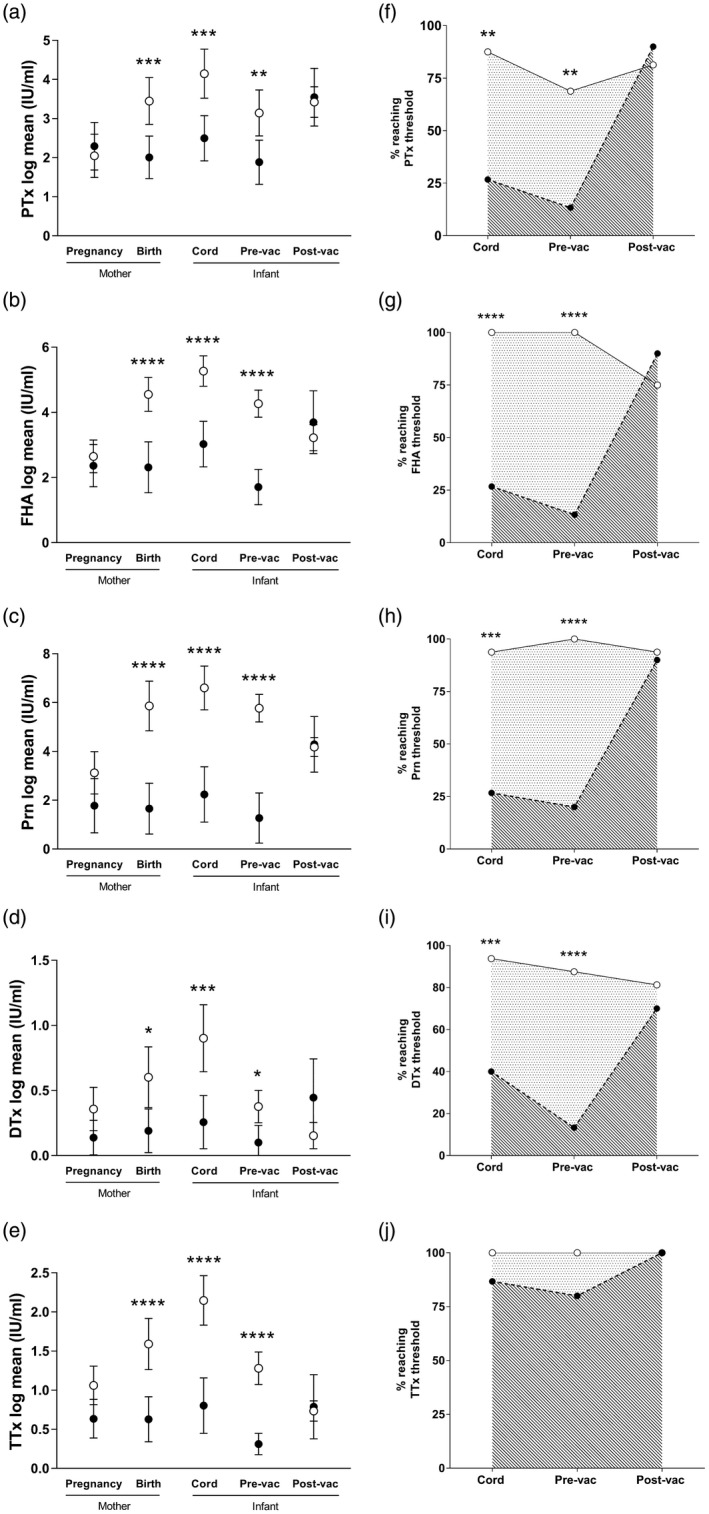
Anti‐tetanus, diphtheria and acellular pertussis (Tdap) antibody concentrations in mothers and their infants from Tdap‐vaccinated and ‐unvaccinated pregnancies (a–e). The proportion of infants from Tdap‐vaccinated and ‐unvaccinated pregnancies reaching antibody thresholds (f–j). (a–e) Anti‐Tdap immunoglobulin (Ig)G were quantified in mother–infant pairs from vaccinated (white circle) and unvaccinated (black circle) pregnancies. Data were log‐transformed, and a random‐effects model applied. Mean and 95% confidence intervals are shown. The vaccinated group had significantly elevated antibodies to (a) PTx, (b) FHA, (c) Prn, (d) Dtx and (e) TTx vaccine antigens in mothers at birth, in cord blood and in the infant prevaccination (pre‐vac; 7 weeks of age). Post‐infant vaccination (post‐vac; 5 months of age), there were no significant differences in antibody to any of the vaccine antigens between vaccinated and unvaccinated groups (*P < 0·05; ***P < 0·001; ****P < 0·0001; unvaccinated *n* = 15; vaccinated *n* = 16). (f–j) Cut‐offs were set at ≥20 IU/ml for pertussis antigens and ≥0·1 IU/ml for DTx and TTx. The proportion of infants at birth, 7 weeks and 5 months that reached these cut‐offs is represented as a percentage of total samples analysed in vaccinated (solid line) and unvaccinated (dashed line) groups. At birth and 7 weeks, the percentage of infants reaching seropositive levels for PTx, FHA, Prn and DTx was significantly higher in the group born to Tdap‐vaccinated mothers than those born to non‐vaccinated mothers. There was no difference for TTx. Post‐primary immunization, there was no difference between the two groups (**P < 0·01; ***P < 0·001; ****P < 0·0001).

At the time of delivery, vaccinated women had significantly higher antibodies against all Tdap vaccine antigens (PTx 72·8 IU/ml; FHA 183·7 IU/ml; Prn 1092·0 IU/ml; DTx 1·16 IU/ml; TTx 5·88 IU/ml) than unvaccinated mothers (PTx 16·3 IU/ml, *P* < 0·001; FHA 83·7 IU/ml, *P* < 0·001; Prn 64·1 IU/ml, *P* < 0·001; DTx 0·15 IU/ml, *P* = 0·01; TTx 1·28 IU/ml, *P* < 0·001) (Fig. 1; for raw data see Supporting information, Table S2). Women in the Tdap‐vaccinated group had higher TTx antibodies at baseline, prior to vaccination (2·23 IU/ml) than those who were not vaccinated (1·14 IU/ml; *P* = 0·02), which was controlled for in the analysis.

### Maternal vaccination is associated with elevated transplacental anti‐DTx and ‐TTx antibody transfer

Ratios between cord and maternal antibody levels at the time of delivery were calculated to measure transplacental antibody transfer. There was positive transport of antibodies to the infant for all Tdap vaccine antigens, independent of vaccination status (Table 1). The transplacental transfer of DTx (2·10) and TTx (2·07) was significantly higher in vaccinated groups compared to unvaccinated groups (DTx 1·64, *P* = 0·04; TTx 1·58, *P* = 0·02).

**Table 1:** Active transfer of tetanus, diphtheria and acellular pertussis (Tdap) vaccine‐specific antibodies from mother to infant. Mean fetal/maternal antibody ratios and 95% confidence intervals (CI) for IgG against Tdap antigens PTx, FHA, Prn, DTx and TTx

Fetal/maternal IgG ratios (CI)			
Vaccine Antigen	Unvaccinated	Vaccinated	*P*‐value
PTx	2·28 (1·16–3·41)	2·16 (1·80–2·53)	0·41
FHA	2·23 (1·53–2·93)	2·15 (1·79–2·51)	0·62
Prn	2·33 (1·07–3·60)	2·14 (1·79–2·69)	0·08
DTx	1·64 (1·42–1·87)	2·10 (1·76–2·46)	0·04
TTx	1·58 (1·35–1·81)	2·07 (1·76–2·39)	0·03

Ig = immunoglobulin; PTx = pertussis toxin; FHA = filamentous haemagglutinin; Prn = pertactin; DTx = diphtheria toxin; TTx = tetanus toxoid.

### Elevated vaccine‐specific antibodies in infants in the first 7 weeks of life after vaccination during pregnancy

Infant blood was collected at birth and 7 weeks, prior to commencement of primary immunization. Infants born to vaccinated mothers had significantly higher antibodies against all Tdap vaccine antigens at birth (PTx 146·5 IU/ml; FHA 342·9 IU/ml; Prn 1899·0 IU/ml; DTx 1·93 IU/ml; TTx 9·99 IU/ml) than infants from unvaccinated mothers (PTx 30·0 IU/ml; FHA 119·1 IU/ml; Prn 134·9 IU/ml; DTx 0·24 IU/ml; TTx 2·04 IU/ml; all *P* < 0·001) (Fig. 1).

At 7 weeks (Fig. 1), infants from vaccinated pregnancies had significantly elevated anti‐PTx (50·4 IU/ml), FHA (111·3 IU/ml), Prn (585·7 IU/ml), DTx (0·55 IU/ml) and TTx (3·00 IU/ml) antibodies compared to infants from unvaccinated pregnancies (PTx 12·9 IU/ml, *P* = 0·004; FHA 12·3 IU/ml, *P* < 0·001; Prn  42·7 IU/ml, *P* < 0·001; DTx 0·05 IU/ml, *P* = 0·01; TTx 0·42 IU/ml, *P* < 0·001). Maternal vaccination had no effect on the half‐life of any of the Tdap antibodies (Table 2).

**Table 2:** Half‐life of tetanus, diphtheria and acellular pertussis (Tdap)‐specific maternal antibody between birth and 7 weeks. Mean half‐life in days and 95% confidence intervals (CI) for maternal IgG against Tdap antigens PTx, FHA, Prn, DTx and TTx

IgG half‐life in infants, in days (CI)			
Vaccine Antigen	Unvaccinated	Vaccinated	*P*‐value
PTx	27·2 (20·0–42·3)	28·9 (26·9–31·3)	0·65
FHA	25·0 (19·5–34·7)	29·7 (27·7–32·1)	0·72
Prn	26·1 (18·5–44·4)	28·1 (25·4–31·3)	0·47
DTx	21·8 (18·2–27·2)	26·1 (24·5–27·9)	0·16
TTx	26·6 (19·3–42·6)	29·5 (26·8–32·8)	0·62

Ig = immunoglobulin; PTx = pertussis toxin; FHA = filamentous haemagglutinin; Prn = pertactin; DTx = diphtheria toxin; TTx = tetanus toxoid.

The percentage of infants reaching protective levels of DTx and TTx antibodies (≥0·1 IU/ml) was calculated based on defined thresholds. Based on other published literature, the percentage of infants reaching an arbitrary threshold of ≥20 IU/ml was reported for the pertussis antigens, as there is no known correlate of protection. At birth and 7 weeks, a significantly higher proportion of infants from Tdap‐vaccinated pregnancies had PTx, FHA and Prn antibodies ≥20 IU/ml and were seroprotected for DTx (≥0·1 IU/ml), compared to unvaccinated pregnancies (Fig. 1). There was no difference in the proportion of infants that were seroprotected for TTx antibody from vaccinated and unvaccinated pregnancies at any time‐point.

### Impact of maternal Tdap vaccination on the infant response to primary pertussis vaccination

To determine the impact of maternal Tdap vaccination on infant responses to pertussis vaccination, blood was collected 1 month after completing their primary course of DTaP‐IPV‐Hib vaccine (8, 12 and 16 weeks). No differences were observed in antibody concentrations, or the percentage of infants reaching defined thresholds to any DTaP vaccine antigens, between infants from vaccinated and unvaccinated pregnancies (Fig. 1). There were no correlations between the concentrations of antibody at birth and in infants post‐primary immunization, either in vaccinated or unvaccinated groups (data not shown).

### Impact of maternal Tdap vaccination on pneumococcal and Hib antibody levels in infants

**Figure 2:**
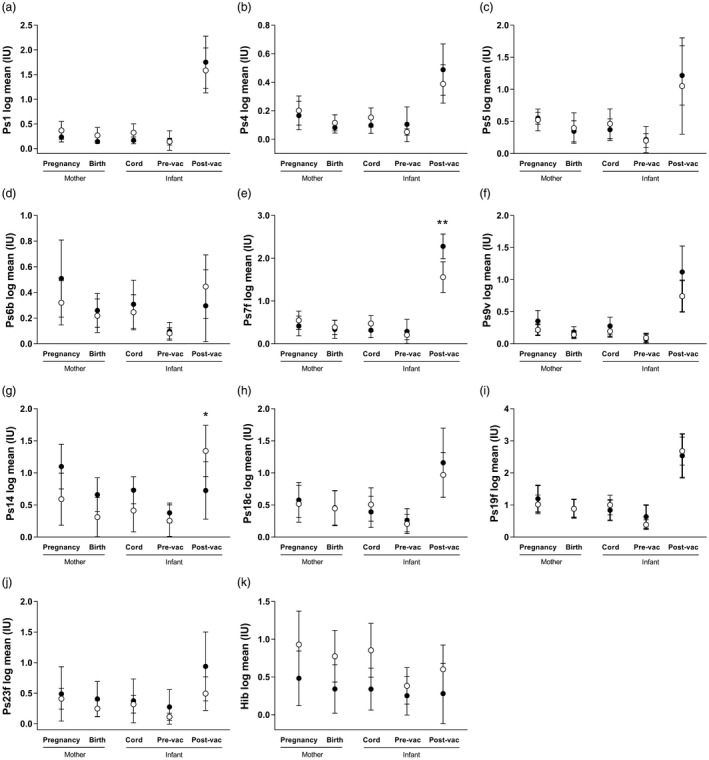
Longitudinal pneumococcal and *Haemophilus influenzae* (Hib) antibody concentrations in mothers and their infants from maternal tetanus, diphtheria and acellular pertussis (Tdap) vaccinated and unvaccinated pregnancies. Immunoglobulin (Ig)G against pneumococcal serotypes (Ps) and Hib were quantified in mother–infant pairs from vaccinated (white circles) and unvaccinated (black circles) pregnancies. Data were log‐transformed, and a random‐effects model applied. Mean and 95% confidence intervals are shown. No differences were observed in antibody to serotypes (a) 1, (b) 4, (c) 5, (d) 6B, (e) 7F, (f) 9V, (g) 14, (h) 18C, (i) 19F, (j) 23F in mothers during pregnancy and at birth, or in cord blood and the infant prevaccination (pre‐vac; 7 weeks of age). Post‐vaccination (post‐vac; 5 months of age), infants from vaccinated pregnancies had elevated serotype 14, whereas infants from the unvaccinated group had elevated 7F. (k) Hib antibody did not differ between vaccinated and unvaccinated groups at any study time‐points (*P < 0·05; **P < 0·01; unvaccinated *n* = 15; vaccinated *n* = 16).

In addition to paediatric DTaP vaccine, potential effects of maternal Tdap vaccination on other vaccines in the primary vaccination schedule were investigated for Hib and pneumococcal polysaccharide serotypes (ST) 1, 4, 5, 6B, 7F, 9V, 14, 18C, 19F and 23F. Pneumococcal and Hib antibody concentrations in 7‐week‐old infants did not differ between infants from vaccinated and unvaccinated pregnancies (Fig. 2). One month after primary PCV13 vaccinations (8 and 16 weeks), infants from unvaccinated pregnancies had significantly higher antibodies against ST7F (8·67 IU/ml, *P* = 0·002), whereas ST14 was increased in infants from vaccinated pregnancies (4·57 IU/ml, *P* = 0·04) (Fig. 2; for raw data, see Supporting information, Table S2). There were no significant differences in antibody to all other antigens between infants born to Tdap vaccinated and unvaccinated mothers, including Hib.

## Discussion

Although the maternal pertussis vaccination programme in the United Kingdom has successfully reduced cases of pertussis in young infants 6, 7, it is important to determine whether increased maternal pertussis antibody in infants is associated with blunted responses to paediatric vaccines. Given that vaccine interference by maternal antibodies has been shown for vaccines such as measles, we determined anti‐pertussis, diphtheria, tetanus, Hib and pneumococcal antibody levels in a prospective cohort of maternally vaccinated and unvaccinated mother–infant pairs. In our small study population, we found that maternal Tdap vaccination results in robust antibody concentrations in mothers and, importantly, in their infants during the critical first weeks of life. With this sample size, we did not detect any significant impact of maternal Tdap vaccination on infant responses to primary pertussis vaccination.

We observed active transplacental transfer of maternal antibodies in Tdap‐vaccinated and ‐unvaccinated groups, as reported by previous studies 15, 16, 18, 25. High levels of maternal antibodies have been linked to reduced transfer ratios, due potentially to saturation of the neonatal Fc receptor (FcRn) for IgG in the placenta 26. We observed no such association, and conversely, transfer of DTx and TTx antibodies were significantly higher in Tdap‐vaccinated pregnancies compared to unvaccinated pregnancies, suggesting that the FcRn is not saturated by the IgG levels induced by maternal vaccination. Maternal antibodies in the infant wane with time, with varying rates reported 15, 27. We report a half‐life of between 25 and 29·7 days, depending on the antigen. Importantly, the half‐life of the antibody from vaccinated pregnancies is the same as that from unvaccinated pregnancies, meaning that antibodies induced by vaccination in pregnancy are just as long‐lasting as antibodies that are present in the mother from previous exposure/vaccination.

Between birth and 7 weeks, infants from vaccinated pregnancies had significantly higher concentrations of antibodies against all acellular pertussis antigens. We used published cut‐offs for tetanus and diphtheria to define protective antibody concentrations 23. No correlate of protection has been defined for pertussis 1; however, high antibody levels are important 28, 29. Several papers have used arbitrary thresholds when analysing pertussis antibody levels 24, 30, 31, and thus for our analysis we utilised the commonly used arbitrary threshold of ≥20 IU/ml for PTx, FHA and Prn antibody concentrations. A significantly higher percentage of infants from vaccinated pregnancies reached these antibody levels for tetanus and diphtheria, and the arbitrarily defined threshold for pertussis antibody in the first 7 weeks of life, compared to infants born from unvaccinated mothers.

Following primary immunization, no differences were observed in concentrations of pertussis antibodies between infants from vaccinated and unvaccinated pregnancies. Although it appears that there is a downward trend in infant FHA, Prn, DTx and TTx antibody levels in the vaccinated group between the 7‐week and 5‐month time‐points, it is important to note that the 7‐week time‐point is essentially a measurement of maternal antibody in infants. As shown by our data, we would expect higher levels of maternal antibodies in infants whose mothers were vaccinated during pregnancy. Unlike in the mothers, it is not possible to compare the pre‐ and post‐vaccination antibody levels in infants to measure their response to pertussis vaccination because of the presence of maternal antibody at the 7‐week time‐point.

In contrast to our findings, a previous larger study in Belgium found that infants from vaccinated pregnancies had lower concentrations of PTx and DTx antibodies following primary immunization, compared to infants from unvaccinated mothers 15. Lower DTx and Prn antibodies have also been observed in infants from Tdap‐vaccinated mothers in Vietnam, compared to a control group whose mothers received a tetanus vaccine during pregnancy 16. The difference between our study and previous studies could be due simply to our limited sample size but also due to women’s vaccination histories, maternal/paediatric vaccine formulations and, in the case of the Vietnamese study, different epidemiological backgrounds, including natural exposure to *B. pertussis*. In the only other study in the United Kingdom, infants from vaccinated pregnancies have previously been shown to have lower PTx, FHA and fimbriae 2/3 antibodies after DTaP vaccination, compared to infants from unvaccinated pregnancies 17. However, the unvaccinated control group in this study was a historical set of infant samples collected 10 months prior to the introduction of the maternal vaccination programme in response to the pertussis outbreak. Thus, these groups could have different confounders, including pertussis exposure, particularly as pertussis prevalence is seasonal 1. In contrast, our study collected samples from vaccinated and unvaccinated pregnancies during the same time‐period.

Pertussis vaccination during pregnancy has been associated with reduced infant responses to other vaccines, such as pneumococcal vaccination 17, 32. We did not observe any differences in the response to Hib vaccination. We also saw very few differences in the concentration of serotype‐specific anti‐pneumococcal antibody between groups; 5‐month‐old infants from unvaccinated pregnancies had elevated ST7F antibodies compared to vaccinated pregnancies and, conversely, vaccinated infants had elevated ST14. However, if we perform Bonferroni correction for the 10 serotypes that were measured, the cut‐off for significance is *P *< 0·005, which the ST7F and ST14 differences do not reach. In contrast to our findings, Ladhani *et al*. 17 and Maertens *et al*. 32 observed blunting of multiple pneumococcal serotypes in infants from Tdap‐vaccinated pregnancies. The blunting of pneumococcal responses was largely removed following a booster vaccination at 12 months of age 32. The reason for the differences between our findings and these studies is not clear, but could be due to our small sample size; continued monitoring of the impact of pertussis vaccination during pregnancy on infant responses to other vaccines is required.

Antibodies produced following acellular vaccination wane rapidly 33. The dynamics of the maternal anti‐pertussis antibody response to vaccination and the efficiency of transplacental antibody transfer rates across gestation need to be considered. In 2016, health authorities in the United Kingdom recommended that maternal pertussis vaccination should be provided earlier in pregnancy, between 16 and 32 weeks of gestation, partly based on evidence that vaccination in the early second trimester (13–25 weeks) resulted in higher cord blood antibody levels than third‐trimester vaccination (≥26 weeks) 34. Earlier vaccination is now also recommended in Ireland, Argentina and Mexico, among others. The extended vaccination window also enables women to be immunized at the time of the 20‐week fetal anomaly scan, potentially increasing the opportunity to administer the vaccine. An additional consideration is preterm birth, which affects 8·6% of births in developed countries 35. Earlier vaccination could also protect these preterm infants 36. We were not able to investigate how the timing of pertussis vaccination impacts on antibody levels. Future studies should analyse the impact of timing of pertussis vaccination during pregnancy and monitor antibody levels between pregnancies to determine whether pertussis vaccination is required with each pregnancy, regardless of the time between pregnancies.

The main limitation of the data presented here is the small number of women and infants with paired samples included: only a small proportion of women agreed to come back for infant follow‐up in observational cohort studies with little tangible benefit for healthy babies. Therefore, our inability to detect potential blunting in vaccine responses in infants born to vaccinated mothers could simply be due to lack of power. We estimated 95% confidence intervals of the effect sizes using bootstrapping, to determine the degree of uncertainty around our estimates. Taking the example of PTx, although we had a very small effect size of –0·1, the bootstrapped confidence interval ranged from –0·78 to 0·66, prohibiting our ability to definitively conclude that there is no difference between the vaccinated and unvaccinated groups. We believe that our study provides useful data for power calculations of future longitudinal mother–infant cohort studies, including the measurement of antibodies against a range of different vaccines (Tdap/DTaP, pneuomoccocal and Hib), antibody half‐life, vaccine interference and the impact of gestation at vaccination.

The UK vaccination schedule at the time of the study meant that both women (Repevax^®^ or Boostrix‐IPV^®^) and infants (Pediacel^®^ or Infanrix‐IPV‐Hib^®^) received one of two vaccines with different antigenic composition. The multiplex assay did not measure FIM antibody contained in both Repevax^®^ and Pediacel^®^. Feunou *et al.* 37 demonstrated in mice that there is the potential for greater interference of maternal antibody when mother–infant pairs receive vaccines from the same manufacturer. We were unable to carry out this type of analysis, given that the majority of women in our study (14 of 16) were vaccinated with Boostrix‐IPV^®^, which does not contain FIM antigen. There is a clinical trial in the United Kingdom to compare antibody levels following maternal vaccination with different Tdap vaccines (ClinicalTrials.gov identifier NCT02145624) that could shed further light on these observations from the mouse model.

## Conclusions

In the United Kingdom, maternal pertussis vaccination during pregnancy protects infants during the critical first weeks of life before commencement of paediatric vaccination, confirming underlying principles for protection of vulnerable newborns against vaccine‐preventable infections via maternal immunization. In this small study, we found that maternal Tdap vaccination results in robust antibody concentrations in infants during their critical first weeks of life, before they receive primary immunization. Future studies should include investigation of the optimal gestation at which to vaccinate pregnant women to maximize high‐vaccine coverage and protection of infants.

## Disclosure

The authors have no conflict of interest to declare.

## Supporting information


**Fig. S1.** Participant flow diagram.

**Table S1**. Demographic data of the MatImms study population included for analysis. Data represent mean values unless stated otherwise, and 95  confidence intervals in parentheses. (NS  =  not significant; NA  =  not applicable).

**Table S2**. Antibody levels against acellular pertussis antigens, Haemophilus influenzae type b and Streptococcus pneumoniae. Untransformed data showing the mean antibody concentrations (IU ml for PTx, FHA, Prn, DTx and TTx; µg ml for Hib and pneumococcal antigens) of all measured antigen specific IgG, at the five study time points in vaccinated and unvaccinated groups. 95  confidence intervals in parentheses, *P* values derived from analysis of log transformed data.
