# Supplementary figures and images for: Antibody responses to Bordetella pertussis and other childhood vaccines in infants born to mothers who received pertussis vaccine in pregnancy – a prospective, observational cohort study from the United Kingdom
Source: Clin Exp Immunol. 2019 Mar 13;197(1):1–10. doi: 10.1111/cei.13275 (PMC6591149; doi:10.1111/cei.13275)

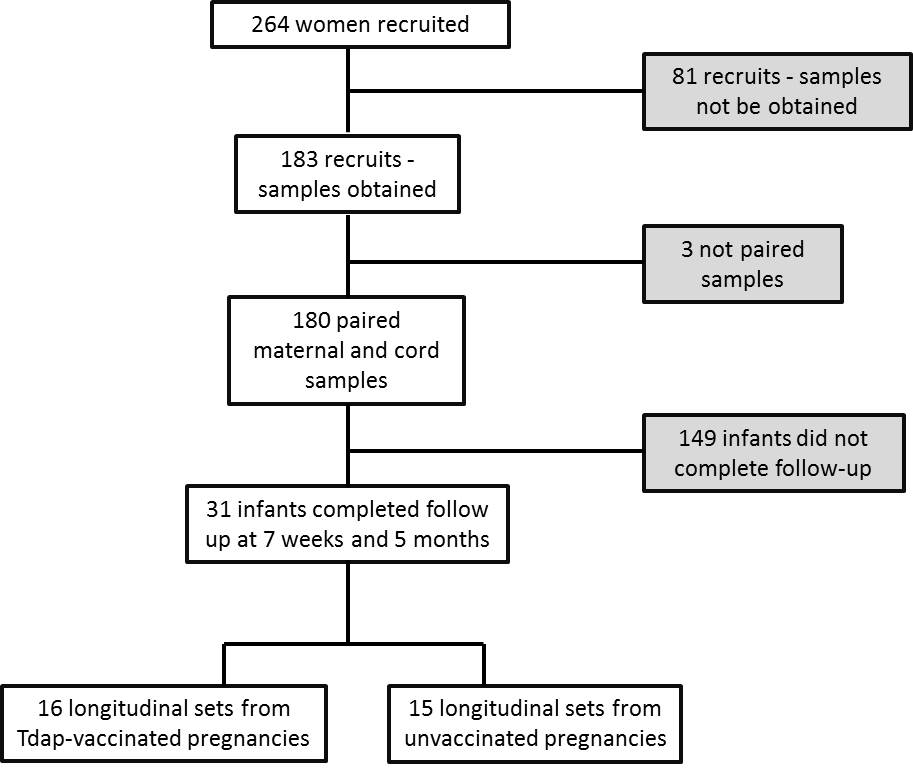

Supplement: Supplementary file 1 — Fig. S1. Participant flow diagram. [file CEI-197-1-s001.jpg]

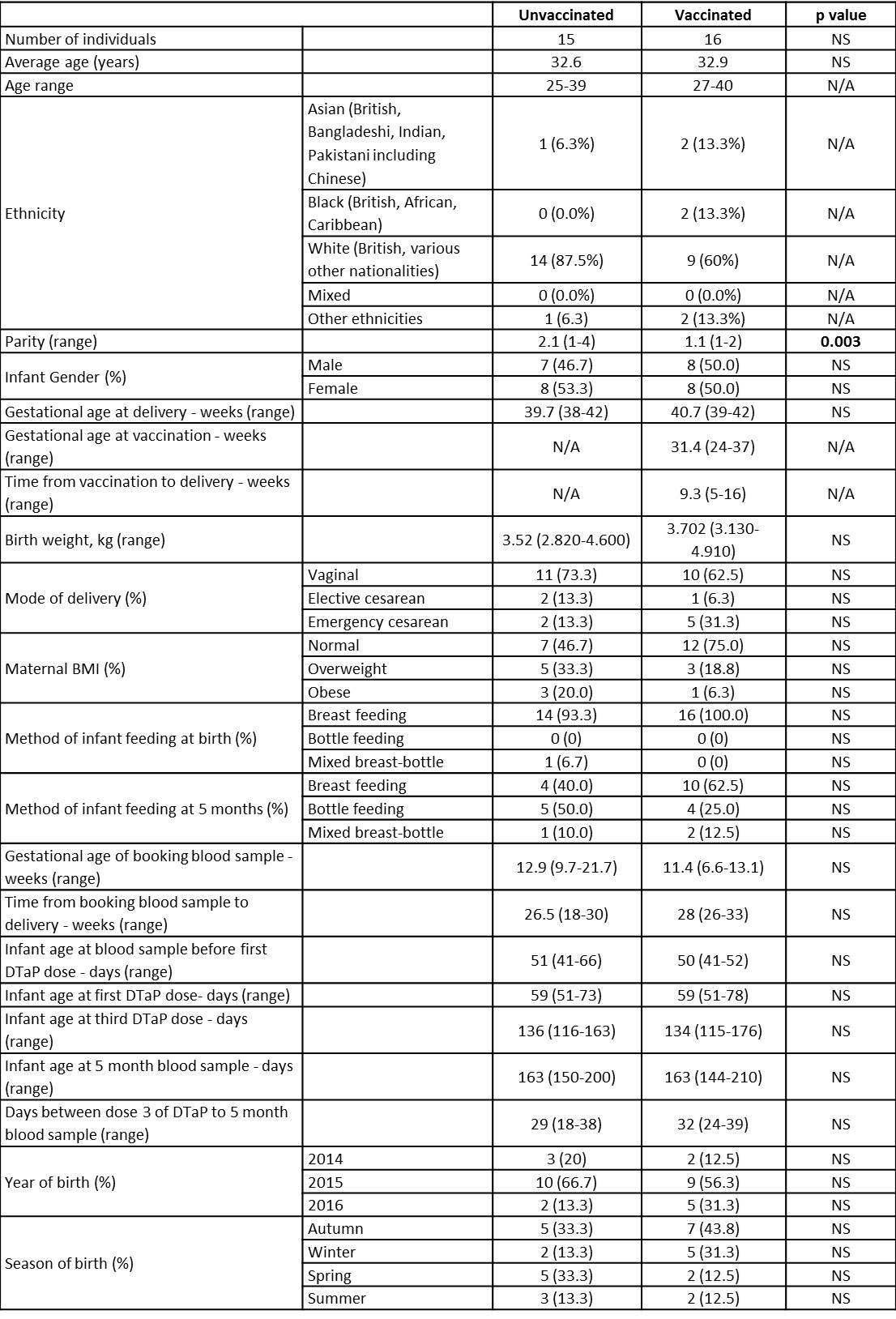

Supplement: Supplementary file 2 — Table S1. Demographic data of the MatImms study population included for analysis. Data represent mean values unless stated otherwise, and 95 confidence intervals in parentheses. (NS = not significant; NA = not applicable). [file CEI-197-1-s002.jpg]

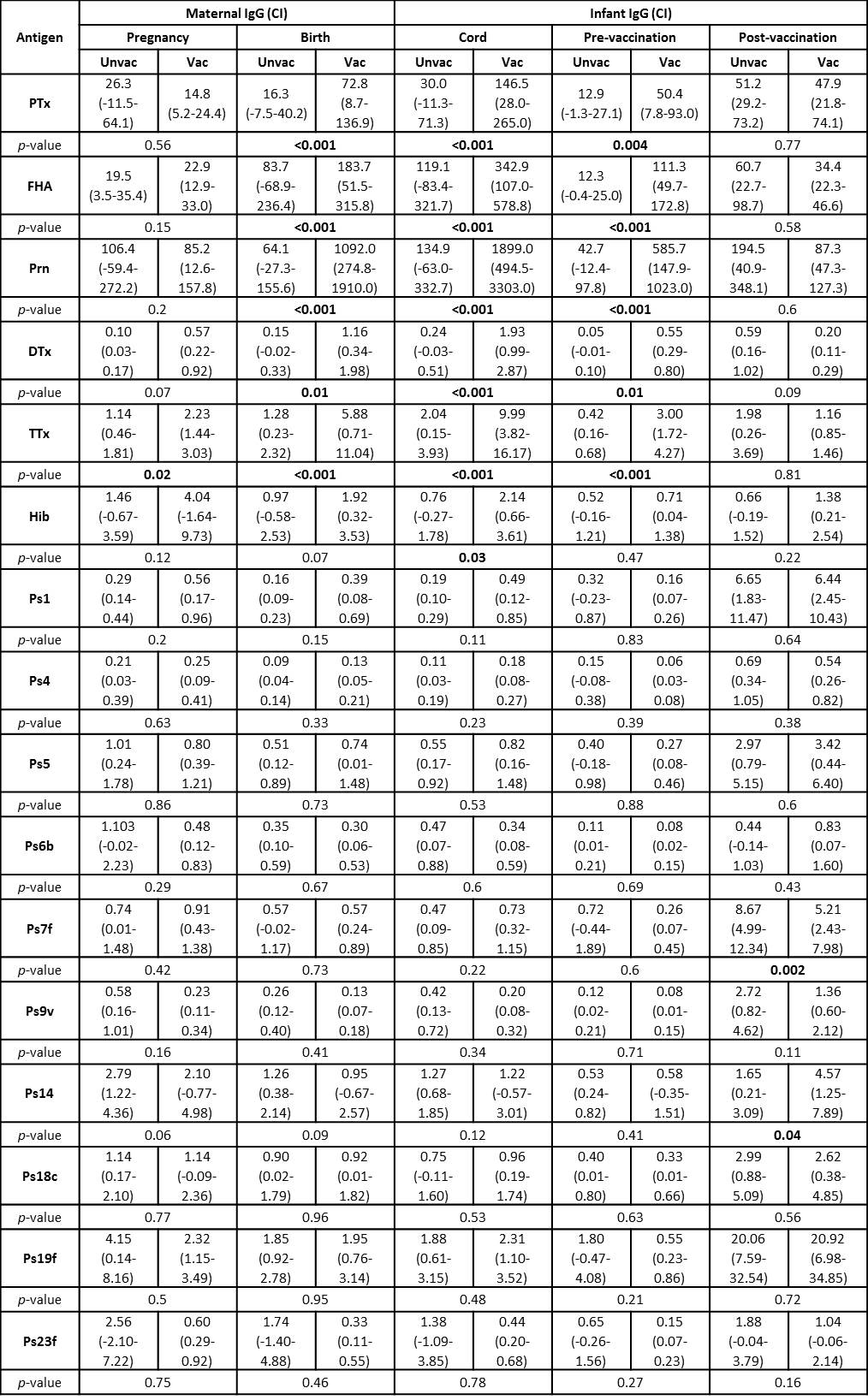

Supplement: Supplementary file 3 — Table S2. Antibody levels against acellular pertussis antigens, Haemophilus influenzae type b and Streptococcus pneumoniae. Untransformed data showing the mean antibody concentrations (IU ml for PTx, FHA, Prn, DTx and TTx; µg ml for Hib and pneumococcal antigens) of all measured antigen specific IgG, at the five study time points in vaccinated and unvaccinated groups. 95 confidence intervals in parentheses, P values derived from analysis of log transformed data. [file CEI-197-1-s003.jpg]
